# Analysis of patient flow and barriers to timely discharge from general medical wards at a tertiary academic hospital in Cape Town, South Africa

**DOI:** 10.1186/s12913-024-10806-6

**Published:** 2024-03-06

**Authors:** Mehreen Hunter, Shrikant Peters, Nontuthuko Khumalo, Mary-Ann Davies

**Affiliations:** 1 Division of Public Health Medicine, School of Public Health, University of Cape Town, Anzio Road, Observatory, Cape Town, 7925 South Africa; 2 Health Intelligence Directorate, Western Cape Government: Department of Health and Wellness, Cape Town, South Africa; 3 Department of Health and Wellness, Groote Schuur Hospital, Cape Town, South Africa

**Keywords:** Patient flow, Patient movement, Discharge planning, South Africa, Healthcare, Efficiency

## Abstract

**Background:**

Movement of patients through a health establishment is a complex activity reliant upon multi-actor co-ordination across departments. The challenge of enhancing service delivery to meet the needs of a growing and aging population, whilst minimizing expense, is a global concern. There is an urgent need to understand and quantify systemic gaps in the efficient delivery of healthcare services. Stagnation of patient flow has negative impacts on both staff and patients by increasing risks of adverse outcomes, staff frustration and job dissatisfaction. An inefficient discharge process can be a significant barrier to timely patient movement.

**Methods:**

A retrospective cohort study was conducted at a tertiary, academic hospital in the Western Cape, South Africa to assess the journey of medical patients from admission to discharge across the five different medical teams (firms) within the general medicine department. Consecutive sampling was used to capture all eligible adult medical in-patients admitted from the emergency department (ED) to general medicine from the 11th – 20th April 2023 and discharged up until the 30th of April 2023.

We reviewed the patient notes (folders) of these individuals using a data-extraction tool to ascertain reasons for admission and barriers to timely discharge.

**Results:**

Among 86 patient folders reviewed, cumulatively accounting for 596 in-patient days, a difference in the median length of in-patient stay between medical firms (*p* = 0.042) was noted. The shortest length of stay corresponded to firms with the greatest proportion of daily senior staff oversight (defined as documented patient reviews by a registrar, medical officer and/or consultant independently or in addition to reviews done for the day by interns and/or students). While 52% of patients vacated their beds between 14:00 and 17:00, 66% of patients were admitted after 20:00. Reasons for prolonged admission were variable, and attributable to a range of different disciplines across the multidisciplinary team.

**Conclusion:**

Whilst this study did not evaluate the appropriateness of chosen medical management but rather systemic drivers affecting patient movement and barriers to timely discharge, the delays in discharge were noted to be multi-factorial including facets across the efficient delivery of medical care, availability of resources and the internal operational frameworks for the institution. Understanding the need to optimize internal process efficiencies with regards to prompt acquisition of investigations, improvement of senior staff oversight and the creation of a standardized discharge process, could enhance efficient patient movement.

## Background

The term “patient flow” describes the enabling process through which patients receive appropriate care, at a suitably designated facility or sub-unit and at the necessary time [1]. “Patient flow management”, however, refers to the facilitation of patient movement within a hospital setting [2]. The complexity of patient flow management is predicated on its reliance upon dynamic, and often, incomplete data, conflicting priorities and the need for multi-actor coordination across departments involved in patient care [2]. Stagnation of patient flow can have severe consequences on both staff and patients including: prolonged patient suffering, healthcare worker burnout, absenteeism, job dissatisfaction and increased medico-legal risk [1, 3].

Globally, healthcare facilities continue to grapple with the complexities of trying to enhance service delivery to meet the needs of a growing and aging population, whilst minimizing expenses [3]. There is, therefore, a need to quantify efficiency in healthcare service delivery, understand the systemic gaps and address deficiencies in an impactful yet sustainable manner.

The discharge process has been identified as a critical barrier to timely patient flow through a hospital system [4]. Delayed discharges can have a domino-effect manifesting in overcrowding of the emergency department, delayed admissions, and delays in inter-departmental referrals, all of which could result in patient dissatisfaction, adverse clinical outcomes and increased expenditure [4]. Factors influencing delayed discharge vary across the literature, but academic medical settings are thought to be particularly affected, due to the shared responsibility of determining the discharge plan between multiple team members (consultants and registrars) and the impact of academic teaching on the efficiency and quality of discharge processes [4]. In 2012, a policy was signed into effect in the Western Cape Province of South Africa to address the growing patient census seeking healthcare at acute facilities [5]. The policy emphasized the need to improve patient throughput by, amongst other recommendations, implementing a discharge process to ensure patient-centredness and continuity of care. Whilst this policy was penned over a decade ago, its relevance has only increased in the post-COVID-19 era where the need to ensure service delivery efficiency and optimal patient movement across the healthcare platform has become even more important in light of the exposed health system challenges to reduce patient dissatisfaction, limit medico-legal risk and safeguard staff wellness.

Perceived contributors to delayed patient discharge include factors that are both intrinsic and extrinsic to the hospital and its staff. Extrinsic factors include the lack of availability of post-acute beds at step-down facilities and delays in patient transport [4]. Intrinsic factors include increased patient numbers, inadequate communication between providers, senior ward round frequency and style, awaiting senior recommendations for care, completion of necessary investigations and a lack of policies and standard operating procedures to guide timely discharges [6–8].

Discharge planning, which can commence from the time of admission, refers to the effective implementation of an individualized discharge plan for patients before they leave the hospital [7]. This practice, which has been adopted in many high-income countries, is done to ensure that patients are discharged on time and have access to sufficient post-discharge support [7]. However, despite the growing evidence in support of discharge planning, many institutions still experience barriers to its implementation. A study conducted in Canada in 2014 [7] sought to describe barriers to patient discharge and identified five themes to this effect: communication challenges between clinicians, between clinicians and other allied health professionals, and between healthcare providers and patients; a lack of role clarity within clinical teams; and deficiency of resources across the healthcare platform; the last two themes identified opportunities for improvement, namely: the need to optimize the structure and function of the medical team through the provision of discharge protocols and targeted ward rounds and, lastly, to identify strong and consistent leadership tasked with coordinating the discharge process.

Early patient discharge is an important consideration within academic facilities [4]. However, it is necessary to recognize that the heterogeneity of healthcare facilities, and teams within facilities, means that they each have their own challenges, stresses, concerns, and priorities. Nevertheless, the consequences of unnecessarily prolonged hospitalizations impeding patient flow has severe effects on the patient, the healthcare provider and hospital facility [1, 3]. As much of the literature on this topic stemmed from high-income countries and studies were largely qualitative in nature, we sought to understand and better quantify the barriers to timely patient discharge within the South African context as fiscal constraints and growing healthcare demands strain our public healthcare system. The aim of this study was to determine the current practices and challenges surrounding patient flow in acute general medicine wards at a tertiary hospital in South Africa. Understanding the factors that influence delayed discharge is necessary to implement targeted interventions that will ultimately improve both the satisfaction and wellness of patients and staff members alike.

## Methods

### Study design and population

A retrospective, observational, cohort patient flow analysis was conducted following the patient journey from their admission to the general medicine division until discharge to identify any barriers to timely egress from the facility. Consecutive sampling was used to capture all acutely ill adult (≥ 18 years) medical in-patients admitted from the emergency unit to general medicine from the 11th – 20th April 2023 and discharged up until the 30th of April 2023.

General medical patients presenting to the Emergency Department (ED) are triaged and assessed by the emergency medical team. They are then treated acutely and either admitted for observation, referred to general medicine or they are discharged. If referred to general medicine, the physician registrar on-call will review the patient and, following a ward round with the consultant, a decision to admit the patient or discharge the patient from care will be made. If admitted, the patient will wait in the ED until a bed becomes available in one of the general medical wards. They will remain in the ward until discharged either back home or successfully accepted to a step-down facility.

Minors (< 18 years), patients who did not begin their inpatient journey at the facility of the study, direct referrals to general medicine from external facilities or other departments within the hospital, patients who were co-managed with other specialist departments, patients that demised during admission and patients who were discharged after the study period were excluded as they would not have undergone the general, expected processes related to patient discharge in the time reviewed and their inclusion could have, therefore, resulted in a skewing of the observed trends.

For the purposes of this study, an in-patient day was included if any part of the 24 h period was spent at the hospital facility. Length of stay, therefore, is not based on specific hourly parameters but is rounded up to the nearest whole day.

### Data collection tool

We used a data extraction form to document the reasons for continued hospital admission, the level of seniority of the reviewing clinician on a daily basis and the admission and discharge times relevant to the patient. For each admission day, only one reason for continued hospital stay was recorded. This was ascertained based on the most pertinent driver behind continued admission. These reasons were based on operational feedback and clinical experience. Whilst some reasons are self-explanatory and relatively straight-forward to deduce based on information provided in the medical notes, others required an interpretation of the clinical picture and a judgement from the researcher based on the information documented for each patient. Some of the reasons requiring an informed judgement by the researcher included attributing ongoing admission to “Awaiting senior review” which was chosen if there was documented clinical improvement, no reason for ongoing admission was discernible and only a junior clinician reviewed the patient. “Delayed discharge process” was selected if any component linked to discharging a patient was cited as a reason for delay (e.g. awaiting discharge medication). If a patient was seen more than once a day, the highest-ranking (most senior) staff member was documented for the purposes of the review. This was determined based on the rank documented in the patient notes and/or through knowledge of the staff members and their rank at the institution. Rank-order was based on the seniority of staff – consultant, registrar, medical officer, intern and medical student. Junior staff members included medical students and interns whilst senior staff members comprised medical officers, registrars and consultants Whilst designed to be a versatile tool able to assist in both real-time and retrospective data capture, the clinical demands of the busy medical unit rendered real-time capturing of data analysis challenging. Therefore, the tool was used to glean information from the folders retrospectively even as patients were admitted into the study upon their presentation to the ED.

This institution houses five medical units/firms. Each medical firm denotes a clinical team headed by different consultants each managing their units according to their individual clinical styles. No firm has an assigned ward, therefore, patients for the firms can be found in any of the designated medical wards. There are five main medical wards that are filled in no particular order. Patients are allocated to the first available bed in any given ward. A sixth, multi-purpose ward is also used for general medical patients and beds are filled according to availability.

As a proxy for the discharge planning process, as no standardized process exists, various factors between medical firms and medical wards were reviewed including: (1) number of patient-days reviewed by each level of clinician; (2) frequency of admission times to wards; and (3) frequency of times that patients vacated ward beds. It must be noted, however, that medical firms and medical wards are not linked and proxy factor 1 speaks to the practices of individual firms whereby factors 2 and 3 speak, predominantly, to the nursing processes in individual wards.

### Data processing and statistical analysis

Data were analysed using Microsoft Excel and RStudio (2023.03.0 + 386) to perform both descriptive and bivariate analyses. Data visualizations were supplemented by DATAtab. Patient characteristics are described in the cohort. We used the Kruskal–Wallis test to assess the differences between the total number of in-patient days between the five medical firms and Chi^2^ tests to assess the associations between the proportion of patients who had prolonged hospital stays, the reasons thereof, and the medical firms where they were admitted.

### Ethical considerations

Approval for conducting this study was obtained from the University of Cape Town’s Human Research Ethics Committee (HREC REF 235/2023) and from the tertiary hospital. A waiver of informed consent was granted by the University of Cape Town’s Human Research Ethics Committee since this study focused on the patient journey rather than sensitive, individual patient information.

## Results

**Fig. 1:**
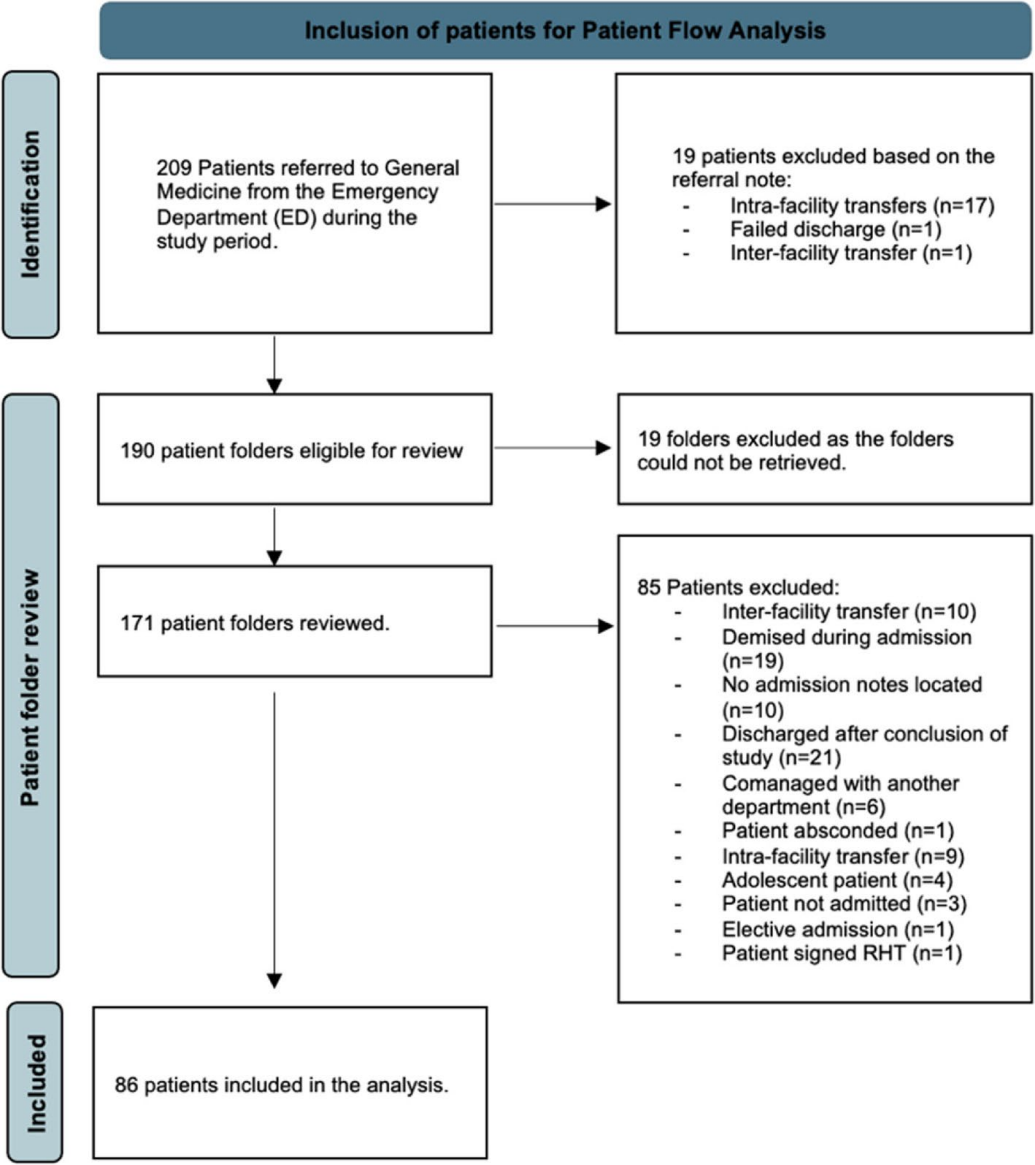
Flowchart denoting the inclusion of patients in the analysis of the study

During the recruitment period, 209 patients were referred to general medicine via the emergency department. Of these, 19 patients were immediately excluded based on the referral note indicating that their patient journey did not begin at their index presentation to the emergency department. Of the remaining 190 patients eligible for review, 19 folders were unable to be retrieved. Therefore, 171 patient folders were reviewed in depth and 85 patients were excluded for various reasons that confounded the patient journey. Eventually, 86 patients were included in the analysis. Figure 1 is a flow chart of patients screened and included in the study.

### Descriptive characteristics of included patients

**Fig. 2:**
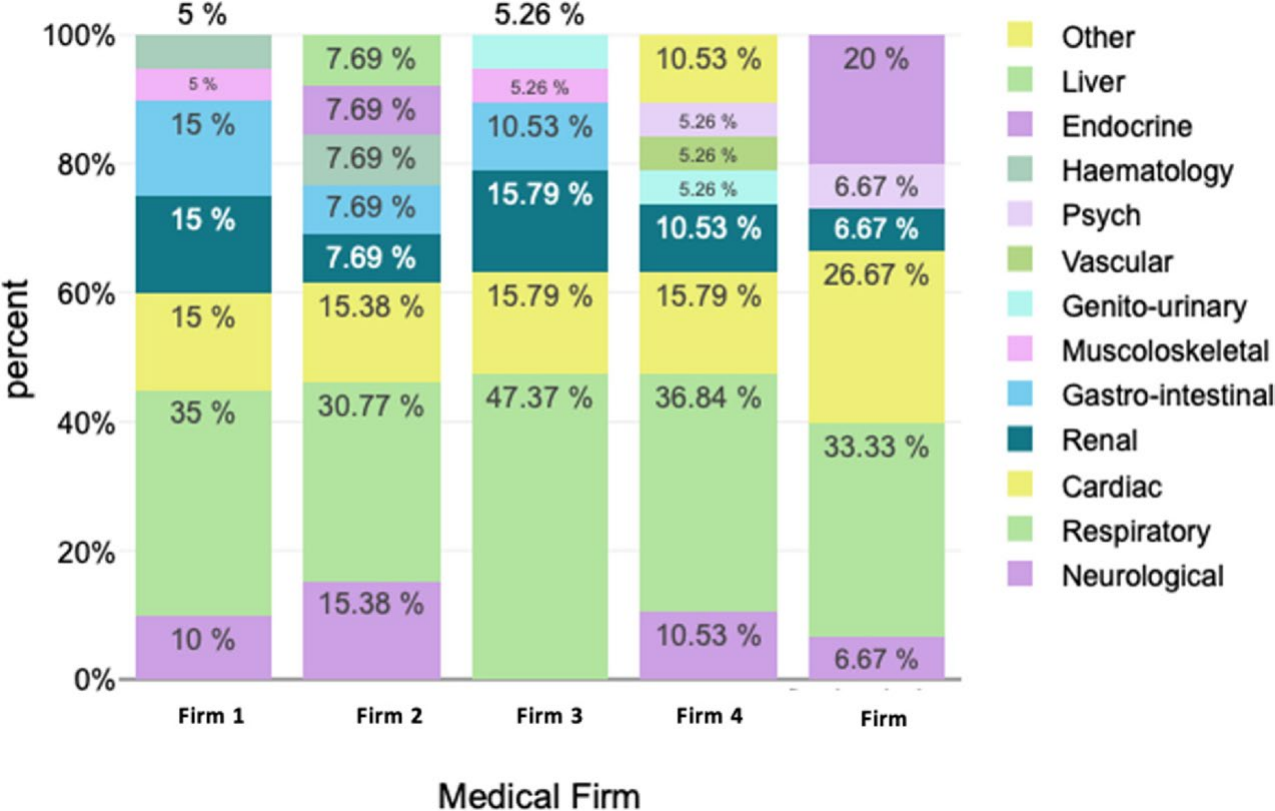
Proportion of diagnostic category seen by each medical firm

Table 1 denotes the characteristics of patients fitting the study criteria, and their distribution across the general medicine platform at the hospital (*n* = 86). The majority of admitted patients were female (59%) and patients were fairly evenly distributed across the five main medical wards – Wards A-D and Ward F – (15 – 20%) with Ward E, expectedly, accounting for only 9% of admitted patients as it is a shared-purpose ward. The frequency of admitted patients across each of the five medical firms was also similar, with Firm 2 accounting for the least number of patients (15%) and Firm 1 accounting for the most (23%). The most common diagnosis category for patient presentation was respiratory diseases (37%) followed by cardiac conditions (17%). This trend was similar across the five medical firms with these two conditions accounting for 46 – 63% of all conditions seen (Fig. 2).

**Table 1:**
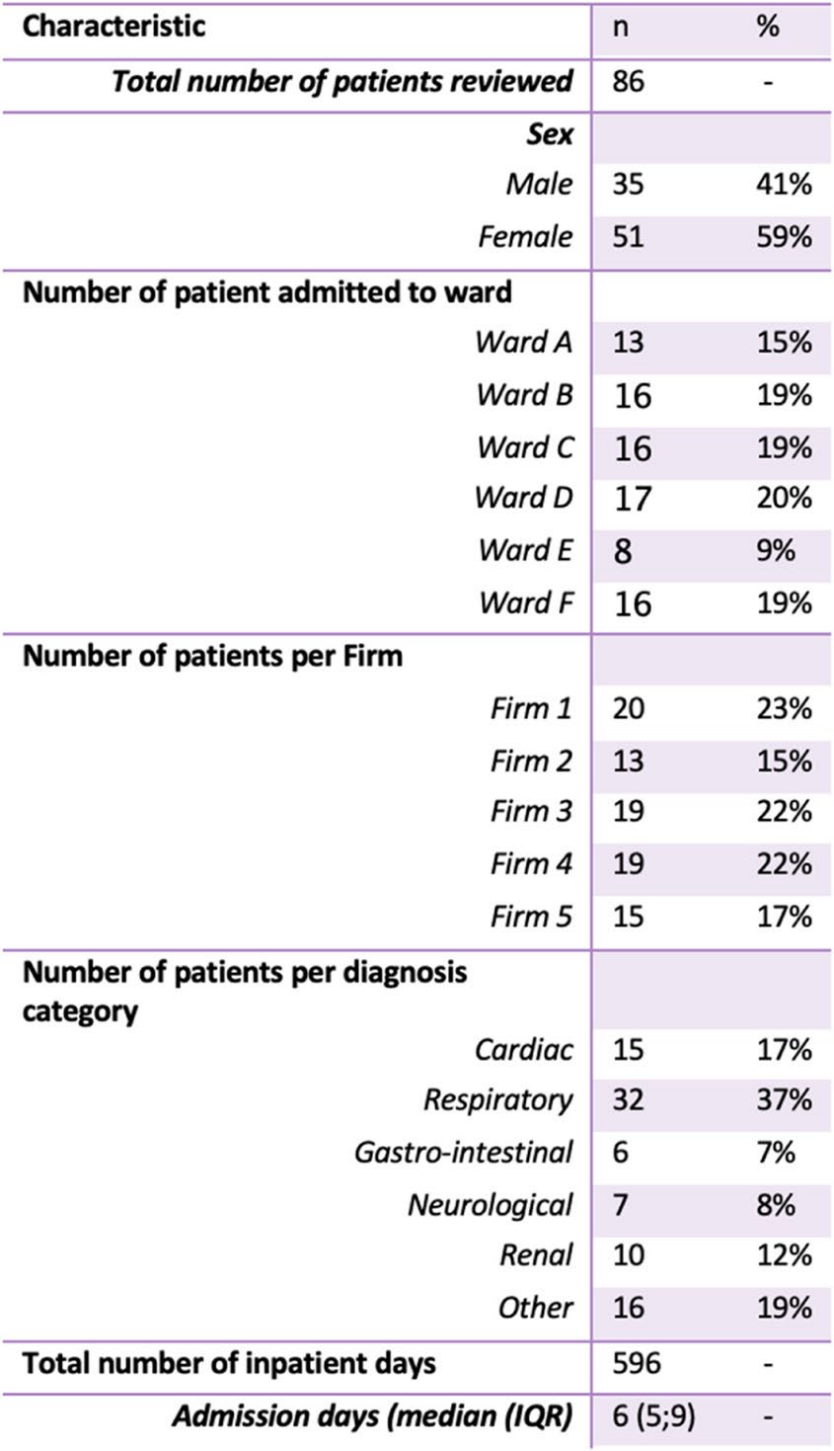
Descriptive characteristics of patients and their distribution across the general medicine platform

### Length of stay per medical firm

**Fig. 3:**
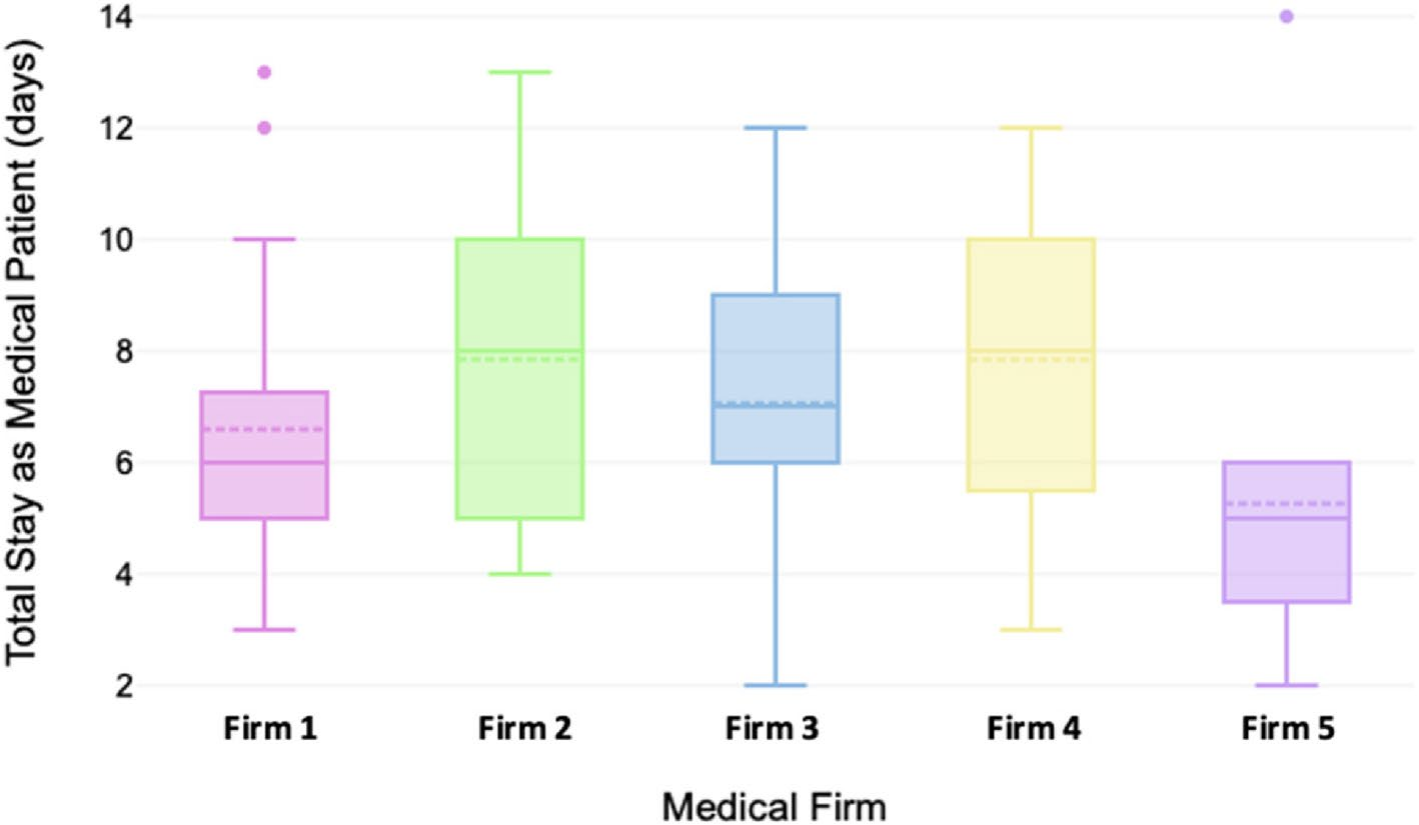
Distribution of inpatient days delineated by firm

**Fig. 4:**
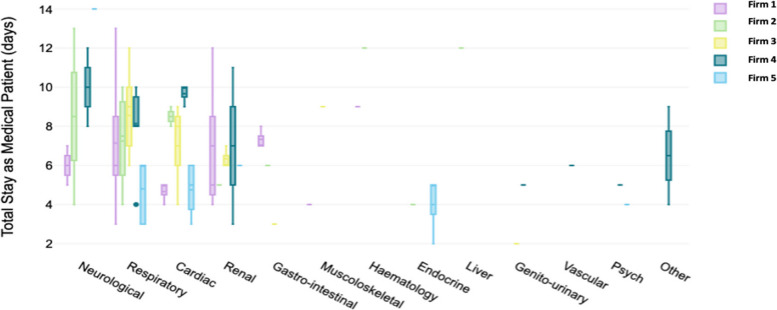
The median length of stay per diagnosis category per medical firm

The Shapiro–Wilk Test revealed a non-normal distribution of patient days across the study period (*p* = 0.007), confirmed by histogram analysis. A total number of 596 in-patient days were included, with an overall median length of stay of 6 (5;9) days per person. The median in-patient stay duration differed across firms (*p* = 0.042) with Firm 5 having the shortest median length of stay and Firms 2 and 4 having the longest length of stay (Fig. 3). However, when considering the variability of diagnostic conditions across firms (Figs. 2 and 4) Firm 5 had the shortest median length of admission for respiratory and cardiac patients relative to the other firms but the longest median admission length for neurological patients, although these comprised a smaller percent of all their admissions relative to most other firms. As most patients seen across all firms were those with cardiac or respiratory illnesses, this could be a contributor to their lower median length of admission.

Overall, 53% of admitted patients during the study could have benefitted from earlier discharge by one or more days. The total number of excess patient-days because of prolonged admission equated to an approximate 15.6% of total admission days (93 out of 596 days).

### Organizational practices and discharge planning

**Fig. 5:**
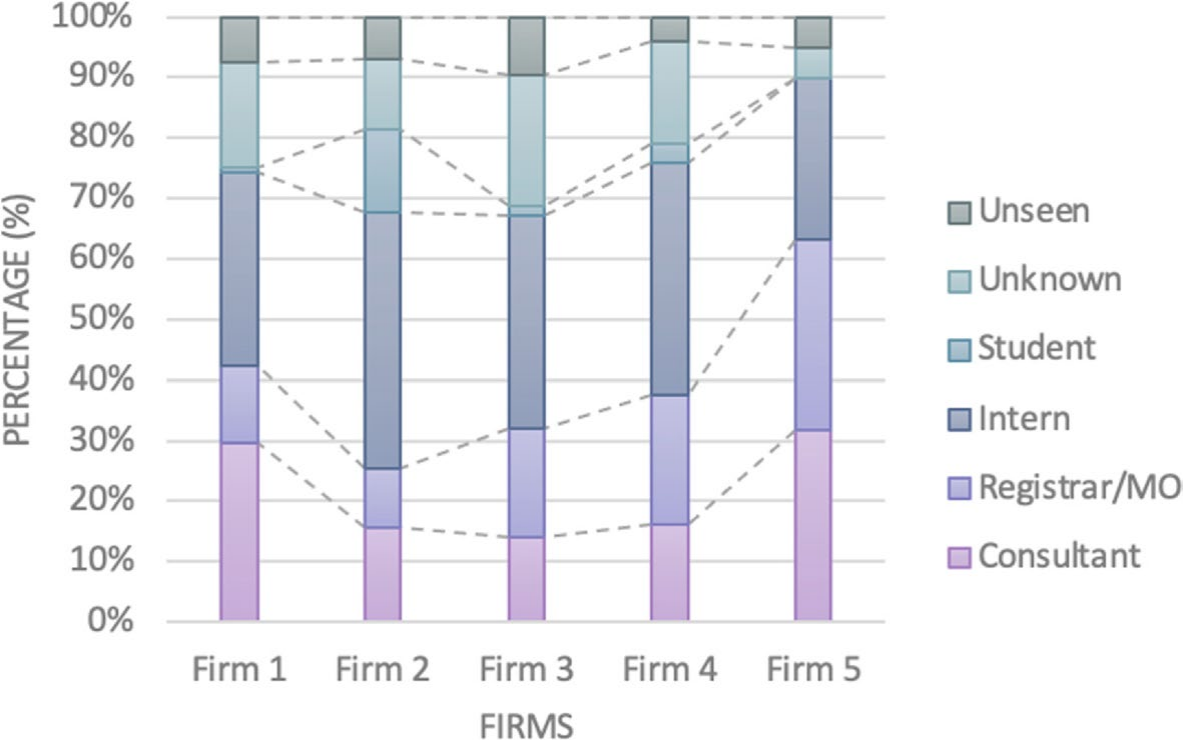
Proportion of patient-days reviewed by each level of staff delineated by firm

Figure 5 highlights that Firm 5 had the highest proportion of senior oversight (consultant and registrar/medical officer review) across the firms (64%) followed by Firm 1 (43%). Firms 3 and 4 had similar proportions of senior oversight at 32% and 37% respectively, whilst Firm 2 had the lowest at 26%. Firm 2 experienced the highest proportion of junior clinician oversight (interns and student reviews) at 56%, followed by Firm 4 (41%). Firm 5 and Firm 1 had the lowest proportions of junior oversight at 27% and 33% respectively.

**Fig. 6:**
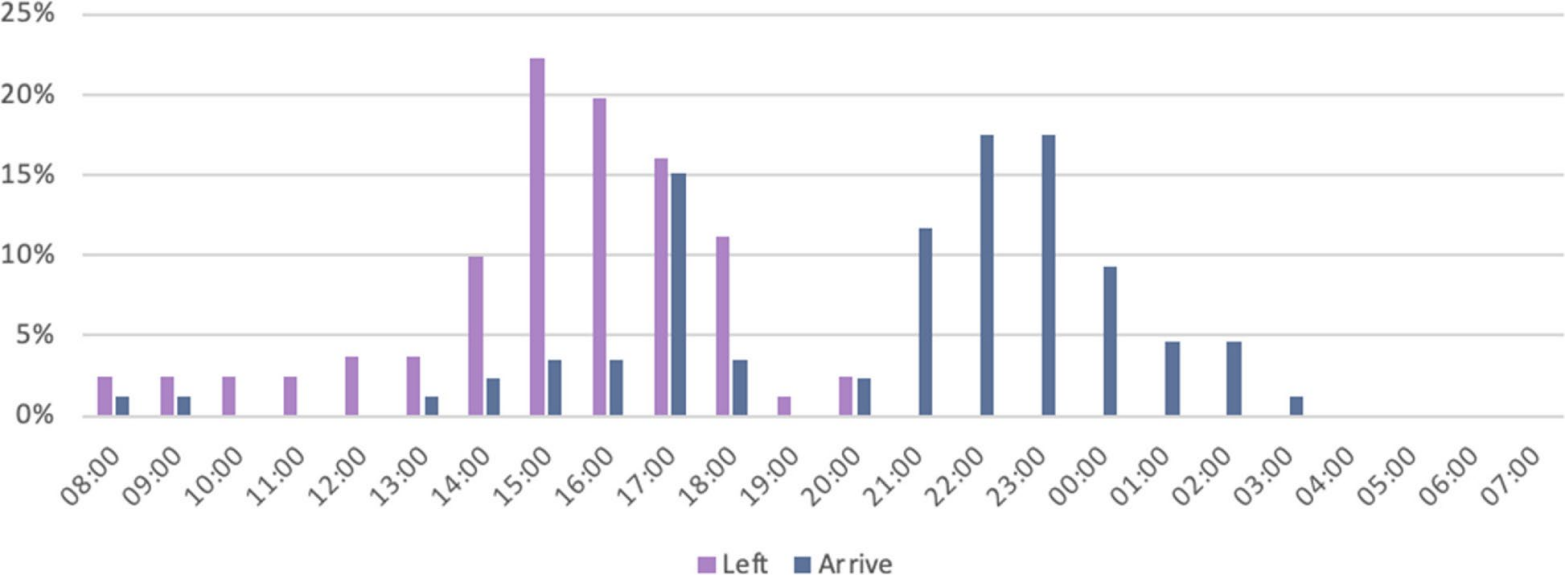
Distribution of times that patients vacated and were admitted to beds during the study period

Figure 6 highlights that more than half (52%) of discharged patients vacated their bed between 14:00 and 17:00. In terms of admission times, almost two-thirds of patients are admitted to wards after 20:00 (66%). There are also time periods of relative inactivity in patient movement in admissions and discharges between 03:00 – 11:00 and 19:00 – 21:00.

### Reasons for prolonged admission

**Fig. 7:**
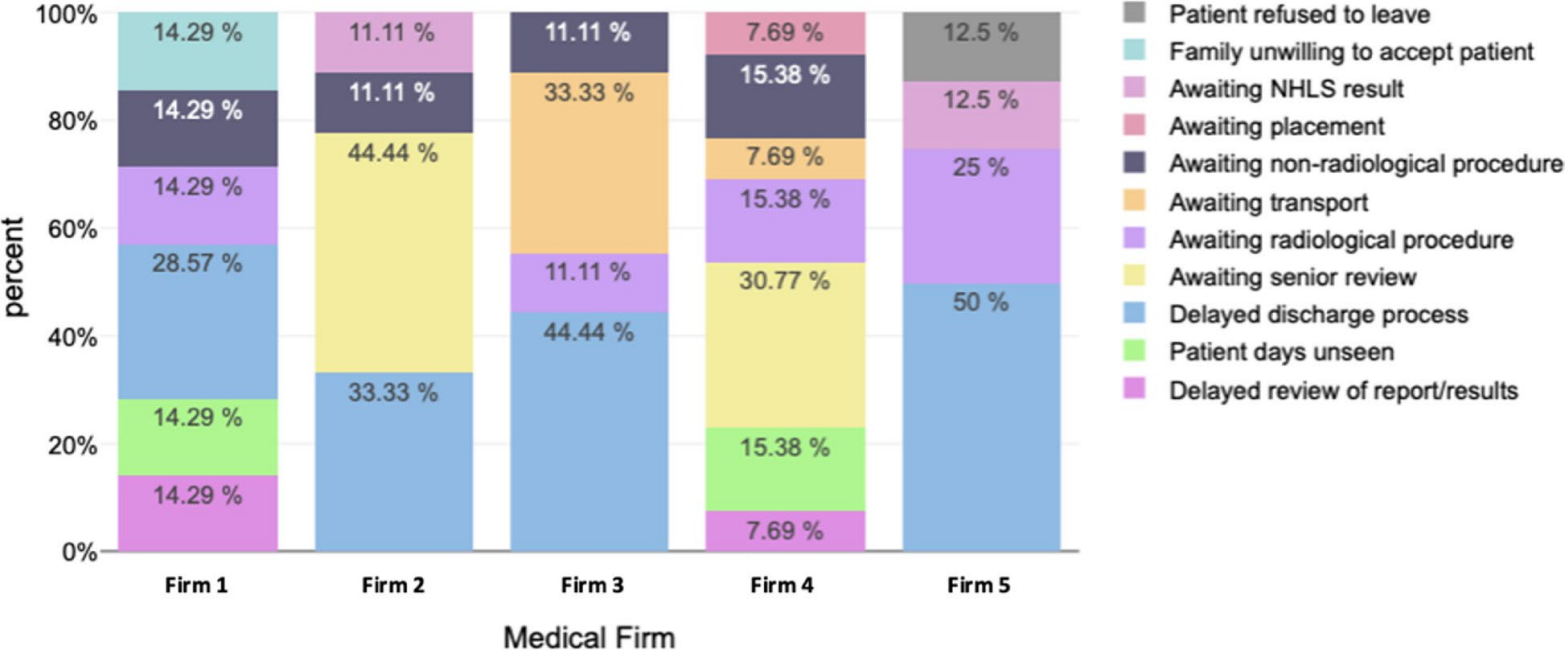
Proportion of patients with prolonged stay by reason per firm

The reasons for admission prolongation were variable, across the multi-disciplinary team. ‘Awaiting radiological procedure’ accounted for the greatest number of excess patient-days at 23%, followed by ‘Awaiting non-radiological procedure’ at 19%, ‘Delayed discharge process’ at 15% and ‘Awaiting Senior Review’ 14% (Table 2). When delineating reasons for prolonged admission by firm (Fig. 7), while the main drivers were quite heterogenous, the differences were not statistically significant (*p*-value 0.13).

**Table 2:**
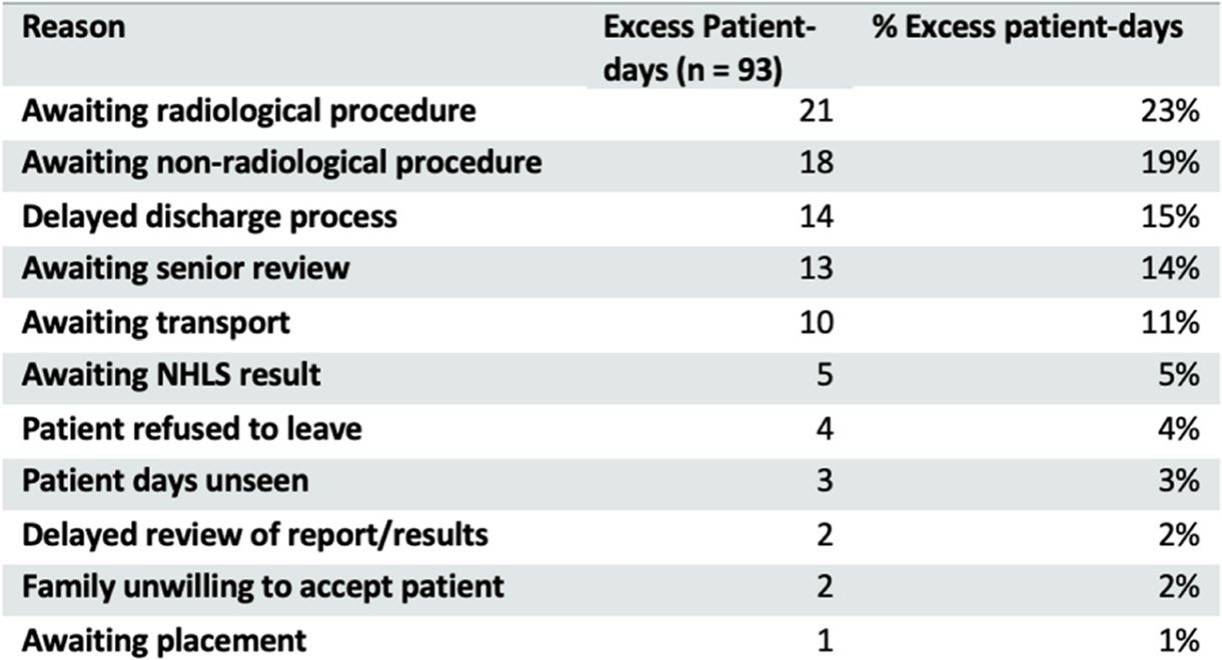
Excess patient-days by reason

## Discussion

The concept of “access block” is well-known amongst the Emergency Medicine fraternity. This is described as a phenomenon whereby patients who have been assessed in the ED and require an inpatient bed, experience delays in moving to the inpatient ward for more than eight hours [9]. Whilst this issue speaks to a whole of health system’s challenge, one contributing aspect is the timely, yet appropriate, discharge of patients from a facility.

Despite this being a small study, it demonstrated significant insights regarding barriers to timely discharge and exposed some of the drivers of stagnation in patient flow. Over half of the patients reviewed in this study could been discharged earlier by one or more days. These prolonged admissions accounted for 15% of the total in-patient days observed. This study did not focus on the financial expenditure linked to patient admission; however, it may be useful to note that at the institution of study, cost per patient-day equivalent (PDE) is R5000.00 (US$264) on average. Therefore, based on this figure, the average excess cost to the institution for the prolonged admissions in this study amounts to R465 000 (US$24 520). Firms with a greater proportion of the most senior (registrars, medical officers and/or consultants) daily reviews, appeared to have shorter median lengths of stay compared to those firms with a reduced proportion of daily senior staff oversight.

The hospital at which this study took place did not have a standardized discharge process, although a provincial policy outlining the need for discharge planning and suggested operational targets was signed into effect in 2012 [5]. Instead, each firm followed its own internal processes to guide in-patient management decision-making, with heterogeneity in clinical and discharge practice. Understanding organizational practices amongst these firms is thus meaningful to determine differences in admission length and their drivers. Our finding that firms with greater senior staff oversight had shorter median patient lengths of stay is in keeping with some of the suggested guidance highlighted in the ‘Emergency Case Load Management Policy for the Department of Health Western Cape’ policy, which recommends an increased frequency in decision-making rounds to increase patient turnover [5]. However, the sample size was too small to properly examine the differences in admission length across the firms for patients with similar diagnoses. The study was also not geared towards evaluating the severity of each patient’s condition. It is possible that the shorter admission lengths observed in Firms 5 and 1 may be due to differences in severity and diagnoses of acute cases relative to the other firms, rather than more efficient patient management.

Review of the times patients leave and are admitted to wards indicates that some of the parameters in the 2012 policy may not be entirely feasible such as the proposal that 90% of patients be discharged by 10:00 on the day they are deemed eligible to leave the hospital. The study outlines that the most common times patients vacate their beds lies between 14:00 – 18:00 (68%), which includes the first visiting hours window of the day (15:00 – 16:00). The most common times for patient admission into ward beds is after 21:00 (66%). There is a notable spike in admissions around 17:00 which, based on operational knowledge of the institution, could be due to continued pressure from the ED and a push from the nursing personnel to conclude admission processes before shift changes at 19:00. Another notable observation is the obvious drop-off in patient movement between 19:00 and 21:00 which corresponds, operationally, with the onset of change in shift as well as includes the second visiting hours window of the day (19:00 – 20:00). This could explain some of the observed trend over these hours. However, as this study did not aim to determine the efficiency of the discharge process in terms of hours but rather focused on the barriers to timely discharge from a system’s perspective causing delays in terms of days, the granular detail to understand why patients vacate beds so late into the day is not possible from these results. Nevertheless, it does expose an area for further research and interrogation to optimize efficiency of patient egress. It also invites further exploration into the push and pull factors associated with “bed-dead time” which refers to the difference in time from when a bed becomes vacant to when it is occupied by a new patient [3].

The finding that majority of patient” (53’) could have benefited from shorter admissions was unsurprising given the intricacies of multi-disciplinary care required in the management of tertiary centre patients. However, it does signal a need for improved efficiency of patient movement, starting with the identification of factors that cause stagnated flow and the fostering of good channels of communication between team-members.

The study revealed that the major drivers for stagnated flow were the awaiting of both radiological and non-radiological procedures (42%). Whilst this invites the institution to review how it manages and prioritizes in-patients for access to these resources as part of its service-delivery requirements across the district’s healthcare platform, it also demonstrates the complexities in managing patient flow, and that not all barriers to efficiency lie within the managing department’s ambit of control. Nevertheless, 29% of excess patient-days were directly attributable to the practices of the medical firms and delays in patient egress or the ‘discharge process’ (writing up the discharge summary, attaining the discharge medication, informing families of the discharge decision, and awaiting collection of the patient). Addressing these factors could reduce excess admission days by up to one-third. As the root causes for stagnated patient flow are multi-factorial, so too should the solutions be, with both admitting and auxiliary teams responsible for improving patient flow through the facility.

### Recommendations

Some of the recommendations, born out of the results of this study, align themselves with those highlighted in the ‘Western Cape Department of Health Emergency Case Load Management Policy’ [5] whilst others are based on knowledge of the institution’s processes:

1. The formation of a discharge plan, developed by the senior clinicians of the firms (consultant) should be done within 24 h of the patient’s admission, and have a clear view of the reason for admission and the parameters required to facilitate discharge once stable.
2. Acknowledging that the patient condition may not be predictable, the reasons for admission should be reviewed daily by the managing team and adjusted where necessary so that all members are aware of the patient plan even in the event of high staff turnover.
3. Senior clinicians must ensure regular review of admitted patients to assist junior professionals with determining eligibility for discharge.
4. Once a patient has been identified for discharge home by the managing clinical team, they should be transferred out of bed to a discharge lounge, whilst awaiting the completion of the relevant paperwork, pharmaceuticals, and transport.
5. Facility processes should seek to prioritize in-patients for clinical support services (e.g., radiology or echocardiograms)
6. Those patients that can reasonably and feasibly receive these services (e.g. radiology or scopes) as an outpatient should be identified early to prevent unnecessarily prolonged admissions.

### Limitations

As this study was conducted over a limited timeframe, and commencing after the Easter long weekend, seasonal and external factors (such as patient behaviour) could have impacted on the results. This duration of this study was relatively short and so some of the prolonged admissions (beyond 14 days) were excluded as their discharge process occurred after conclusion of the study. Therefore, some reasons for stagnated flow, i.e., ‘Awaiting Placement’ may be underrepresented in these results. The study included a small number of eligible participants which could have resulted in it being underpowered. Even though this was a retrospective study in terms of folder reviews, patients for inclusion were recruited in real-time meaning that staff were aware that the study was being conducted. Additionally, for ethical purposes, a declaration of this study was made available in each general medical ward to alert staff and patients to the ongoing nature of this study. This could have altered staff behaviour and standard practice patterns via the Hawthorne Effect. Whilst designed to accommodate both prospective and retrospective data collection, the busyness of the medical units resulted in the data capture being undertaken solely retrospectively. This could have introduced a form of missing data bias as the investigator could only glean information from the accuracy and robustness of the notetaking, without having a full understanding of all aspects of the patient’s clinical course, including pertinent points pertaining to discharge which may not have been documented. Furthermore, to determine the reason for ongoing admission, as the review of data was retrospective, a judgement call needed to be made by the researchers which had to be done based on the available data in the notes. The clinical practice of individual clinicians (conservative versus aggressive) was also not explicitly explored as patient admission lengths were seen as a proxy marker for this. Furthermore, for a substantial proportion of patient-days across the firms the level of staff reviewing the patient was “unknown” owing to the staff member and/or their rank being indiscernible in the patient notes. This could have impacted on the results obtained but also highlights the importance of good patient record-keeping. Further study is needed to better understand the relative contributions of senior oversight and clinical practice style to patient length of stay independent of diagnosis and disease severity.

The results may not be generalizable to other public facilities at higher or lower levels of care either within or external to the Western Cape Province, given the unique organizational practices, patient profile and structure of the hospital. The calculation of “length of stay” for the purposes of this study are also not entirely aligned with the National Indicator Data Set (NIDS) as only a specific subset of patients was reviewed for the study and the full spectrum of separations (deaths and transfers out) were not factored into the analysis. Additionally, the results of this analysis may not be generalizable to hospitals in the private sector as only practices from a singular, public sector, tertiary academic facility were observed.

## Conclusion

The need to enhance the efficiency of patient-flow through hospital facilities is paramount to ensure optimal wellness of staff and patients alike. This study provides a better understanding of some of the challenges to timely patient movement and provides recommendations to improve some of the current operational practices. Improving operational efficiency and use of resources through ensuring timely attainment of both radiological and non-radiological procedures and their results for in-patients, developing a standardized discharge planning process, improving senior oversight and inter as well as intra-departmental communication could assist in improving patient flow. Continued monitoring, auditing, and research into the granular causes of stagnated patient flow are necessary for continued improvement.

## Data Availability

The de-identified datasets used for analysis in the study are available to reviewers from the corresponding author upon reasonable request.

## Abbreviations

HREC: Human Research Ethics Committee
OHRP: US Office for Human Research Protections
ED: Emergency Department
PDE: Patient-day Equivalent
NHLS: National Health Laboratory Service
